# Handles of Plugging Instruments

**Published:** 1856-12

**Authors:** 


### HANDLES OF PLUGGING INSTRUMENTS.

Though the kind of handles we have to our excavators and plugging instruments would seem to be a small matter, yet it is a very important one that they *have* handles, and that they be the right kind. I fear many of us are in the habit of overlooking small matters, or those which we view as small things. Or we are like those *few very fortunate* individuals, who are so exceedingly familiar with all these minute *practical points*, that a single moment's thought bestowed upon them would be a work of supererogation on *our* part.

In an interview with Dr. Rich, of New York, a short time since, he showed us some plugging instruments with handles superior in some respects, to anything we had before seen in that line.

The shaft of the instrument is first prepared from the square or round cast steel rod of the proper size; if round it is roughened. The remaining portion of the handle, or rather the covering, for this is of thick leather, prepared, put on and finished in the following manner: Round pieces of leather are cut as large in diameter as the handle is desired, a hole is punched in the centre of each just large enough to admit the rod or shaft, already prepared. Enough of these are placed on the shaft to form the handle, and then glued altogether and to the shaft. As soon as the glue is put on a clamp with holes through it large enough to receive the shaft, is put on, and the pieces pressed together as firmly as possible. After it is perfectly dried, the clamp is removed and the handle dressed with a file, or turned, which would be better, and made perfectly smooth. They can be made of any desired form.

Some advantages in these handles are at first view apparent. They can be made as large as desired with less weight than of ivory, ebony or steel. They have a soft velvety touch, and far less liable to slip in the hand than either of the others, and require less grasp to hold them.

This is a method by which any one may supply himself with handles, and in form just such as he may wish.

Dr. Rich has been experimenting in this line. He remarked that he had tried many materials for handles, and this was the best substance he had yet found.

We intend to have some handles made in this way, try them, and report further.

